# Supplementary material for: Prognostic Impact of Immunoglobulin Kappa C (IGKC) in Early Breast Cancer
Source: Cancers (Basel). 2021 Jul 20;13(14):3626. doi: 10.3390/cancers13143626 (PMC8304855; doi:10.3390/cancers13143626)
Supplement: Supplementary file 1 [file cancers-13-03626-s001.zip › cancers-1259930-supplementary.pdf]

cancers

MDPI

Supplementary Materials: Prognostic Impact of Immunoglobulin Kappa C (*IGKC*) in Early Breast Cancer

Marcus Schmidt, Karolina Edlund, Jan G. Hengstler, Anne-Sophie Heimes, Katrin Almstedt, Antje Lebrecht, Slavomir Krajnak, Marco J. Battista, Walburgis Brenner, Annette Hasenburg, Jörg Rahnenführer, Mathias Gehrmann, Pirkko-Liisa Kellokumpu-Lehtinen, Ralph M. Wirtz and Heikki Joensuu

Table S1. Cox univariable analysis for DDFS including all patients ($n = 909$). CI, confidence interval; IGKC, immunoglobulin kappa C; HER2, human epidermal growth factor receptor 2; TNBC, triple-negative breast cancer.

Variable	Hazard ratio	95% CI	*p*
IGKC, continuous	0.982	0.920–1.048	0.589
IGKC			
< median	1.000		
≥ median	0.948	0.685–1.312	0.748
IGKC			
< 75th quantile	1.000		
≥ 75th quantile	0.862	0.585–1.270	0.453
Age			
≤ 50 years	1.000		
> 50 years	0.829	0.599–1.147	0.258
ER status			
Negative	1.000		
Positive	0.522	0.375–0.728	<0.001
PR status [1]			
Negative	1.000		
Positive	0.667	0.482–0.922	0.014
HER2 status			
Negative	1.000		
Positive	1.744	1.233–2.469	0.002
Molecular subtype			
Luminal	1.000		
TNBC	2.354	1.545–3.587	<0.001
HER2+	2.143	1.476–3.112	<0.001
Ki67 [2]			
≤ 20%	1.000		
> 20%	1.984	1.383–2.847	<0.001
pT stage [1]			
pT1	1.000		
pT2-4	1.748	1.230–2.484	0.002
pN stage			
pN0	1.000		
pN1-3	2.149	1.053–4.382	0.035
Grade [3]			
Grade I	1.000		
Grade II-III	4.331	1.911–9.814	<0.001

[1] $n = 908$, [2] $n = 809$, [3] $n = 869$

Table S2. Cox multivariable analysis for DDFS (all patients). 868 patients had complete data for all variables and were included in the multivariable analysis. *IGKC* expression was dichotomized using the median **(a)** or the top quartile **(b)** as cutoff. The results for *IGKC* expression as a continuous variable are shown in Table 2. CI, confidence interval; IGKC, immunoglobulin kappa C; HER2, human epidermal growth factor receptor 2; TNBC, triple-negative breast cancer.

(a)

**Variable**	**Hazard Ratio**	**95% CI**	***p***
IGKC			
< median	1.000		
≥ median	0.815	0.580–1.145	0.238
Age			
≤ 50 years	1.000		
> 50 years	0.899	0.643–1.257	0.533
Molecular subtype			
Luminal	1.000		
TNBC	2.467	1.557–3.909	<0.001
HER2+	2.007	1.355–2.972	0.001
pT stage			
pT1	1.000		
pT2-4	1.622	1.123–2.343	0.010
pN stage			
pN0	1.000		
pN1-3	4.175	1.908–9.135	<0.001
Grade			
Grade I	1.000		
Grade II-III	2.834	1.219–6.592	0.016

(b)

**Variable**	**Hazard Ratio**	**95% CI**	***p***
IGKC			
< 75th quantile	1.000		
≥ 75th quantile	0.619	0.410–0.934	0.022
Age			
≤ 50 years	1.000		
> 50 years	0.904	0.646–1.263	0.553
Molecular subtype			
Luminal	1.000		
TNBC	2.689	1.690–4.279	<0.001
HER2+	2.087	1.410–3.090	<0.001
pT stage			
pT1	1.000		
pT2-4	1.604	1.111–2.316	0.012
pN stage			
pN0	1.000		
pN1-3	4.322	1.974–9.464	<0.001
Grade			
Grade I	1.000		
Grade II-III	2.979	1.282–6.924	0.011

Table S3. Cox univariable and multivariable analyses for DDFS in patients with TNBC. Median *IGKC* expression for the patients with TNBC **(a)** or the top quartile **(b)** was used as a cut-off to distinguish between low and high *IGKC*. 132 patients had data for IGKC and were included in the univariable analysis. 129 patients had complete data for all variables and were included in the multivariable analysis. CI, confidence interval; IGKC, immunoglobulin kappa C; HER2, human epidermal growth factor receptor 2

(a)

	**Univariable Analysis**			**Multivariable Analysis**		
**Variable**	**Hazard ratio**	**95% CI**	***p***	**Hazard ratio**	**95% CI**	***p***
IGKC						
< median	1.000			1.000		
≥ median	0.418	0.198–0.882	0.022	0.322	0.146–0.712	0.005
Age						
≤ 50 years	1.000			1.000		
> 50 years	0.427	0.209–0.874	0.020	0.324	0.150–0.701	0.004
pT stage						
pT1	1.000			1.000		
pT2-4	0.839	0.388–1.813	0.654	1.469	0.630–3.427	0.373
pN stage						
pN0	1.000			1.000		
pN1-3	2.796	1.076–7.262	0.035	3.932	1.321–11.705	0.014
Grade [1]						
Grade I-II [2]	1.000			1.000		
Grade III	0.642	0.286–1.442	0.283	0.735	0.319–1.693	0.470

[1] $n = 129$; [2] grade was dichotomized as I-II vs III since there was only one TNBC patient with a grade I tumor.

(b)

	**Univariable Analysis**			**Multivariable Analysis**		
**Variable**	**Hazard ratio**	**95% CI**	***p***	**Hazard ratio**	**95% CI**	***p***
IGKC						
< 75th quantile	1.000			1.000		
≥ 75th quantile	0.172	0.041–0.719	0.016	0.197	0.045–0.852	0.030
Age						
≤ 50 years	1.000			1.000		
> 50 years	0.427	0.209–0.874	0.020	0.465	0.216–1.003	0.051
pT stage						
pT1	1.000			1.000		
pT2-4	0.839	0.388–1.813	0.654	1.193	0.510–2.789	0.684
pN stage						
pN0	1.000			1.000		
pN1-3	2.796	1.076–7.262	0.035	3.527	1.179–10.550	0.024
Grade [1]						
Grade I-II [2]	1.000			1.000		
Grade III	0.642	0.286–1.442	0.283	0.782	0.337–1.813	0.566

[1] $n = 129$; [2] grade was dichotomized as I-II vs III since there was only one TNBC patient with a grade I tumor.

Table S4: Cox univariable and multivariable analysis for DDFS in patients with luminal breast cancer. 574 patients had data for *IGKC* and were included in the univariable analysis. 540 patients had complete data for all variables and were included in the multivariable analysis. *IGKC* expression was included as a continuous variable **(a)** or the median **(b)** or top quartile **(c)** of *IGKC* expression in patient with luminal breast cancer was used as a cut-off to distinguish between low and high *IGKC*. CI, confidence interval; IGKC, immunoglobulin kappa C; HER2, human epidermal growth factor receptor 2

(a)

	Univariable Analysis			Multivariable Analysis		
**Variable**	**Hazard ratio**	**95% CI**	***p***	**Hazard ratio**	**95% CI**	***p***
IGKC	0.968	0.876–1.070	0.524	0.960	0.868–1.061	0.419
Age						
≤ 50 years	1.000			1.000		
> 50 years	0.920	0.569–1.490	0.735	1.135	0.685–1.880	0.623
pT stage [1]						
pT1	1.000			1.000		
pT2-4	2.454	1.454–4.142	0.001	2.228	1.285–3.865	0.004
pN stage						
pN0	1.000			1.000		
pN1-3	3.171	0.440–22.847	0.252	4.208	0.574–30.832	0.157
Grade [2]						
Grade I	1.000			1.000		
Grade II-III	3.793	1.521–9.459	0.004	3.241	1.284–8.183	0.013

[1] $n = 573$, [2] $n = 541$

(b)

	Univariable Analysis			Multivariable Analysis		
**Variable**	**Hazard ratio**	**95% CI**	***p***	**Hazard ratio**	**95% CI**	***p***
IGKC						
< median	1.000			1.000		
≥ median	1.000	0.619-1.614	0.999	0.970	0.592–1.592	0.906
Age						
≤ 50 years	1.000			1.000		
> 50 years	0.920	0.569–1.490	0.735	1.137	0.686–1.884	0.618
pT stage [1]						
pT1	1.000			1.000		
pT2-4	2.454	1.454–4.142	0.001	2.246	1.296–3.895	0.004
pN stage						
pN0	1.000			1.000		
pN1-3	3.171	0.440–22.847	0.252	4.257	0.581–31.182	0.154
Grade [2]						
Grade I	1.000			1.000		
Grade II-III	3.793	1.521–9.459	0.004	3.234	1.280–8.169	0.013

[1] $n = 573$, [2] $n = 541$

(c)

	Univariable Analysis			Multivariable Analysis		
**Variable**	**Hazard ratio**	**95% CI**	***p***	**Hazard ratio**	**95% CI**	***p***
IGKC						
< 75th quantile	1.000			1.000		
≥ 75th quantile	0.634	0.339–1.184	0.153	0.578	0.301–1.109	0.099
Age						
≤ 50 years	1.000			1.000		
> 50 years	0.920	0.569–1.490	0.735	1.138	0.687–1.884	0.615
pT stage [1]						
pT1	1.000			1.000		
pT2-4	2.454	1.454–4.142	0.001	2.249	1.298–3.895	0.004
pN stage						
pN0	1.000			1.000		
pN1-3	3.171	0.440–22.847	0.252	4.309	0.589–31.538	0.150
Grade [2]						
Grade I	1.000			1.000		
Grade II-III	3.793	1.521–9.459	0.004	3.305	1.308–8.349	0.011

[1] n = 573, [2] n = 541

Table S5: Cox univariable and multivariable analysis for DDFS in patients with HER2-positive breast cancer. 203 patients had data for *IGKC* and were included in the univariable analysis. 199 patients had complete data for all variables and were included in the multivariable analysis. *IGKC* expression was included as a continuous variable **(a)** or the median **(b)** or top quartile **(c)** of *IGKC* expression in HER2-positive patients was used as a cut-off to distinguish between low and high *IGKC*. CI, confidence interval; IGKC, immunoglobulin kappa C; HER2, human epidermal growth factor receptor 2

(a)

	**Univariable Analysis**			**Multivariable Analysis**		
**Variable**	**Hazard ratio**	**95% CI**	***p***	**Hazard ratio**	**95% CI**	***p***
IGKC	0.944	0.837–1.064	0.343	0.933	0.826–1.055	0.271
Age						
≤ 50 years	1.000			1.000		
> 50 years	1.159	0.650–2.067	0.616	1.219	0.675–2.201	0.512
pT stage						
pT1	1.000			1.000		
pT2-4	1.053	0.576–1.926	0.867	1.272	0.688–2.351	0.442
pN stage						
pN0	1.000			1.000		
pN1-3	4.322	1.048–17.820	0.043	4.863	1.155–20.479	0.031
Grade [1]						
Grade I-II [2]	1.000			1.000		
Grade III	0.817	0.452–1.477	0.504	0.910	0.501–1.653	0.757

[1] $n = 199$; [2] grade was dichotomized as I-II vs III since there was only five HER2-positive patients with a grade I tumor.

(b)

	**Univariable Analysis**			**Multivariable Analysis**		
**Variable**	**Hazard ratio**	**95% CI**	***p***	**Hazard ratio**	**95% CI**	***p***
IGKC						
< median	1.000			1.000		
≥ median	0.688	0.386-1.226	0.204	0.730	0.406–1.315	0.295
Age						
≤ 50 years	1.000			1.000		
> 50 years	1.159	0.650–2.067	0.616	1.230	0.680–2.225	0.494
pT stage						
pT1	1.000			1.000		
pT2-4	1.053	0.576–1.926	0.867	1.269	0.687–2.344	0.447
pN stage						
pN0	1.000			1.000		
pN1-3	4.322	1.048–17.820	0.043	4.584	1.091–19.258	0.038
Grade [1]						
Grade I-II [2]	1.000			1.000		
Grade III	0.817	0.452–1.477	0.504	0.930	0.510–1.696	0.813

[1] $n = 199$; [2] grade was dichotomized as I-II vs III since there was only five HER2-positive patients with a grade I tumor.

(c)

	**Univariable Analysis**			**Multivariable Analysis**		
**Variable**	**Hazard ratio**	**95% CI**	***p***	**Hazard ratio**	**95% CI**	***p***
IGKC						

< 75th quantile	1.000			1.000		
≥ 75th quantile	0.949	0.527–1.709	0.862	0.910	0.500–1.656	0.757
Age						
≤ 50 years	1.000			1.000		
> 50 years	1.159	0.650–2.067	0.616	1.208	0.669–2.183	0.531
pT stage						
pT1	1.000			1.000		
pT2-4	1.053	0.576–1.926	0.867	1.264	0.684–2.336	0.455
pN stage						
pN0	1.000			1.000		
pN1-3	4.322	1.048–17.820	0.043	4.718	1.118–19.907	0.035
Grade [1]						
Grade I-II [2]	1.000			1.000		
Grade III	0.817	0.452–1.477	0.504	0.909	0.499–1.655	0.754

[1] $n = 199$; [2] grade was dichotomized as I-II vs III since there was only five HER2-positive patients with a grade I tumor.
